# Supplementary material for: Hybrid Models and Biological Model Reduction with PyDSTool
Source: PLoS Comput Biol. 2012 Aug 9;8(8):e1002628. doi: 10.1371/journal.pcbi.1002628 (PMC3415397; doi:10.1371/journal.pcbi.1002628)
Supplement: Text S4 — Complete source code for the PyDSTool package (version 0.88.120504). Includes API documentation and help files linking to web pages. This file is identical to the current public release on Sourceforge.net. (ZIP) [file pcbi.1002628.s004.zip › PyDSTool/html/toc-PyDSTool.Toolbox.optimizers.step-module.html]

xml version="1.0" encoding="ascii"?


step


# Module step

---

## Classes

GoldfeldStep  
 GoldsteinPriceStep  
 GradientStep  
 LocalBruteForce1DStep  
 MarquardtStep  
 NewtonStep  
 PartialStep  
 RestartNotOrthogonalConjugateGradientStep  
 RestartPeriodicallyConjugateGradientStep  

## Functions

CWConjugateGradientStep  
 DConjugateGradientStep  
 DYConjugateGradientStep  
 FRConjugateGradientStep  
 FRPRPConjugateGradientStep  
 PRPConjugateGradientStep  

## Variables

step\_\_all\_\_

---

[hide private]
